# Supplementary material for: The crystal structure of Thermus thermophilus UMP kinase complexed with a phosphoryl group acceptor and donor
Source: PLoS One. 2025 Sep 2;20(9):e0330398. doi: 10.1371/journal.pone.0330398 (PMC12404492; doi:10.1371/journal.pone.0330398)
Supplement: Attachment — Submitted filename: Response ToReviewers.docx [file pone.0330398.s003.docx]

Comments to the Editor

PLOS ONE

**PONE-D-25-12007R2**

The crystal structure of *Thermus thermophilus* UMP kinase complexed with a phosphoryl group acceptor and donor

We found the academic editor's comments most helpful. We revised the manuscript with considering reviewers' comments as follows. The texts after ==> are our comments on the revision.

1. Please include a legend for figure 6.

==> We added the legend to Fig. 6.
